# Systematic Review of Antimicrobial Therapies and Clinical Outcomes in *Pseudomonas aeruginosa* Infections

**DOI:** 10.3390/life16081267

**Published:** 2026-07-31

**Authors:** Silvijus Abramavičius, Dalia Akramienė, Tashfeen Tashfeen, Dovilė Abramavičienė, Edgaras Stankevičius

**Affiliations:** 1 Preclinical Research Laboratory for Medicinal Products, Institute of Cardiology, Lithuanian University of Health Sciences, A. Mickevičiaus g. 9, LT-44307 Kaunas, Lithuania; silvijus.abramavicius@lsmu.lt (S.A.); edgaras.stankevicius@lsmu.lt (E.S.); 2 Institute of Physiology and Pharmacology, Lithuanian University of Health Sciences, LT-44307 Kaunas, Lithuania; dalia.akramiene@lsmu.lt; 3 Pharmacological Evolution Laboratory, Department of Biochemistry, Quaid-I-Azam University, Islamabad 45320, Pakistan; tashfeen@bs.qau.edu.pk

**Keywords:** *Pseudomonas aeruginosa*, antimicrobial therapy, antimicrobial resistance, clinical outcomes, pharmacokinetics/pharmacodynamics (PK/PD), systematic review, meta-analysis

## Abstract

*Pseudomonas aeruginosa* is an important opportunistic pathogen associated with diverse healthcare-associated infections and increasing antimicrobial resistance. This systematic review evaluated antimicrobial treatment regimens, clinical outcomes, and pharmacokinetic/pharmacodynamic (PK/PD) considerations in *P. aeruginosa* infections. A systematic review was conducted according to PRISMA guidelines. PubMed/MEDLINE and the Cochrane Library were searched for studies published between January 2000 and March 2026. Data on antimicrobial therapies, clinical and microbiological outcomes, and PK/PD parameters were extracted. Study quality was assessed using the NHLBI quality assessment tool. Narrative synthesis was performed for all eligible studies, and a quantitative meta-analysis was conducted for studies with standardized outcome data. Of the 982 records identified, 249 studies met the inclusion criteria, and 34 were included in the quantitative meta-analysis. The evidence base was highly heterogeneous regarding study design, patient populations, infection types, and treatment regimens. Respiratory infections, particularly in cystic fibrosis patients, accounted for most studies. Quantitative synthesis showed no consistent superiority of any antimicrobial regimen (or combination of them), while treatment outcomes were influenced by infection characteristics, antimicrobial resistance patterns, and PK/PD factors. No universally superior antimicrobial regimen was identified for *Pseudomonas aeruginosa* infections. Some novel and promising treatment options have been identified, such as anti-*Pseudomonas aeruginosa* LPS monoclonal antibody panobacumab and inhaled Clostridium butyricum delivered via oxygen-driven nebulization. Two novel treatment modalities did not yield a clinically relevant effect, namely, the bispecific monoclonal antibody MEDI3902 (gremubamab) and the IC43 *Pseudomonas aeruginosa* vaccine. Treatment effectiveness appears to depend on clinical context, antimicrobial susceptibility, and PK/PD considerations, highlighting the need for further standardized research.

## 1. Introduction

*Pseudomonas aeruginosa* is a ubiquitous Gram-negative opportunistic pathogen and the most frequent cause of acute and chronic infections, particularly in immunocompromised patients and patients with underlying comorbidities. It causes respiratory tract infections, bloodstream infections, urinary tract infections, and device-associated infections, and is among the most prevalent pathogens of healthcare-associated infections in the world [1,2]. *P. aeruginosa* in patients with cystic fibrosis (CF) turns into a chronic colonizer of the lungs, contributing to morbidity, the gradual loss of lung functionality, and death [2,3]. The fact that it is a severe hazard on the list of the priority pathogens of the World Health Organization also explains the topicality of its implementation in clinical practice due to antimicrobial resistance [4].

The ability of *P. aeruginosa* to resist antibiotics in various ways is a great peculiarity that contributes to the failure of treatment, persistence of infection, and lack of therapy options, especially when it is multidrug-resistant (MDR) and extensively drug-resistant (XDR), and is largely due to efflux pump expression, reduced permeability of the outer membrane, target site mutation, and antibiotic degradation [5].

Despite the existence of certain anti-pseudomonal agents like beta-lactams, fluoroquinolones, aminoglycosides, and polymyxins, the clinical outcome is not foreseeable, and the most adequate options for treatment are not so obvious. The same efficacy of different antimicrobial regimens is frequently suggested by comparative studies, and treatment is frequently chosen depending on local resistance patterns, severity of the infection, and patient-specific factors rather than being demonstrated to be more effective [6,7]. In addition, the increasing resistance to last-line agents, including carbapenems and polymyxins, has raised the need to provide personalized and tailored care.

Pharmacodynamic and pharmacokinetic (PK/PD) factors have become increasingly important in antimicrobial therapy. Adequate exposure of the drug relative to bacterial susceptibility is often expressed in terms of PK/PD indices of time above minimum inhibitory concentration (T>MIC) with beta-lactams or area under the curve to minimum inhibitory concentration ratio (AUC/MIC) with beta-lactams, which are also important to maximize efficacy and minimize resistance development [8]. The problem of tissue penetration changes, especially in biofilm-associated infections, also troubles the effective use of antimicrobials.

To date, the evidence base is highly heterogeneous, and the nature of studies, the nature of infections, and their outcomes of interest are all variable despite the numerous studies that evaluated the antimicrobial treatment of *P. aeruginosa*. In most of these studies, the target is narrowed down to a specific group of individuals, e.g., CF patients, and may not be applicable in other clinical settings. Nor is there any standardization of the incorporation of PK/PD into clinical consequences, nor is there a giant synthesis as yet that would encompass microbiological, pharmacological, and clinical viewpoints.

These limitations should include a systematic review of the available evidence to contribute to the description of the effectiveness of antimicrobial therapy for *Pseudomonas aeruginosa* infections. The outcomes of the treatment vary, despite numerous studies on the topic, and there is no definite comparative effectiveness of treatment options. Therefore, this systematic review will focus on exploring antimicrobial treatment regimens and their clinical effects in various infections and patients. Moreover, relevant pharmacodynamics and pharmacokinetics are also addressed as the mechanisms that lead to the reaction to treatment. The present review is targeted at summarizing the existing evidence from heterogeneous study designs and clinical settings, and the identification of the gaps that should be filled in future studies to inform the most suitable patient management and treatment.

## 2. Materials and Methods

### 2.1. Study Design

This systematic review was conducted in accordance with the Preferred Reporting Items of Systematic Reviews and Meta-Analyses (PRISMA) guidelines. The review protocol was not prospectively registered in PROSPERO or any other systematic review registry. This represents a limitation of the review because prospective protocol registration enhances methodological transparency and reduces the potential risk of reporting bias. Nevertheless, the review methodology, including the eligibility criteria, search strategy, study selection, data extraction, quality assessment, and statistical analyses, was predefined before the review was conducted and was applied consistently throughout the study. This review was aimed at assessing antimicrobial treatment regimens and clinical outcomes in patients with *Pseudomonas aeruginosa* infections.

The review question was formulated using the PICO framework as follows: Population (P): Patients with *Pseudomonas aeruginosa* infections. Intervention (I): Systemic or inhaled antimicrobial therapies. Comparator (C): Alternative antimicrobial regimens, combination therapies, placebo, or standard care. Outcome (O): Clinical outcomes, microbiological eradication, mortality, pharmacokinetic/pharmacodynamic (PK/PD) parameters, and treatment-related adverse events.

### 2.2. Information Sources

The MEDLINE database through PubMed and the Cochrane Library were used for the systematic literature search. Moreover, all included articles were screened through the reference lists of the included articles to identify additional potentially relevant studies that were not initially identified in the database search.

### 2.3. Search Strategy

The search strategy was formulated to find studies that evaluated antimicrobial treatment for *Pseudomonas aeruginosa* infections. The main search term was *Pseudomonas aeruginosa*, which was searched in the title and abstract fields.

A systematic literature search was conducted to identify relevant studies on *Pseudomonas aeruginosa* infections and antimicrobial treatment outcomes. The search was performed in MEDLINE (via PubMed), the Cochrane Library, and the reference lists of all included studies were manually screened to identify additional eligible publications.

To improve comprehensiveness and reduce publication bias, the search strategy was expanded to include additional databases: Embase, Web of Science, and Scopus. These databases were selected due to their broader biomedical and pharmacological coverage, particularly for clinical trials and pharmacokinetic/pharmacodynamic studies.

#### 2.3.1. Search Terms and Strategy

The core search term used across all databases was

- “*Pseudomonas aeruginosa*”

This term was adapted using database-specific syntax and combined with Boolean operators and controlled vocabulary (e.g., MeSH terms in PubMed where applicable). The full search string used in PubMed was structured as follows:

- PubMed example search string:

(“*Pseudomonas aeruginosa*”[Title/Abstract] OR “*Pseudomonas aeruginosa*”[MeSH Terms])

AND

(infection*[Title/Abstract] OR treatment*[Title/Abstract] OR therap*[Title/Abstract] OR antimicrobial*[Title/Abstract] OR antibiotic*[Title/Abstract])

AND

(“pharmacokinetic*”[Title/Abstract] OR “pharmacodynamic*”[Title/Abstract] OR outcome*[Title/Abstract])

The search strategy was adapted for each database using the appropriate controlled vocabulary and database-specific syntax. In PubMed, MeSH terms were used where applicable, whereas Emtree terms were used in Embase. Equivalent keyword combinations, Boolean operators (AND/OR), truncation symbols, and database-specific field tags were applied in Web of Science, Scopus, and the Cochrane Library. Searches were restricted to human studies published between January 2000 and March 2026 in English, German, or French.

#### 2.3.2. Filters and Limits

Search results were restricted using the following filters:

- Language: English, German, and French.
- Publication type: Clinical trials, randomized controlled trials, meta-analyses, and systematic reviews. Published systematic reviews and meta-analyses were screened only to identify additional eligible primary studies through their reference lists and were not included in the qualitative or quantitative evidence synthesis.
- Study population: Human studies only.
- Publication years: only studies published in the period between 1 January 2000 and 31 March 2026, were included in the search.
- Database filters: Applied where available (e.g., PubMed Clinical Trial, RCT, Systematic Review filters, Cochrane review filters).

##### Additional Search Methods

To ensure completeness, reference lists of all included studies and relevant reviews were manually screened for additional eligible publications not captured in the database search. Compliance with the PRISMA-S reporting checklist is summarized in Table 1.

### 2.4. Inclusion and Exclusion Criteria

For the studies included in the quantitative meta-analysis, additional selection criteria were applied. Studies were included if they reported standardized clinical and/or microbiological outcomes, and the necessary numerical data were available. Studies eligible for the quantitative meta-analysis were selected from the 249 studies included in the systematic review based on predefined methodological criteria. Specifically, studies were required to report standardized and comparable clinical and/or microbiological outcomes, provide sufficient numerical data for effect size calculation (including treatment and comparator group data), and evaluate comparable antimicrobial interventions. Studies with heterogeneous outcome definitions, insufficient quantitative data, duplicate populations, or designs unsuitable for statistical pooling were included only in the narrative synthesis. As a result, 34 studies met all eligibility criteria for quantitative synthesis and were included in the meta-analysis.

#### 2.4.1. Inclusion Criteria

Studies that compared the systemic antimicrobial therapy (intravenous (IV), oral (PO), or inhalation (INH)) were included in the quantitative synthesis. Included studies were required to report clinical outcomes such as clinical cure, clinical success, or marked improvement in infection-related signs and symptoms at specified time points of evaluation (e.g., Test of Cure). Microbiological outcome measures for the eradication or presumed eradication of baseline pathogens were also summarized. Studies involving both paediatric and adult populations were eligible for inclusion if they met the predefined eligibility criteria. These populations were included in the qualitative synthesis to provide a comprehensive overview of the available evidence. For the quantitative meta-analysis, studies were pooled only when they reported comparable interventions, outcome definitions, and sufficient quantitative data, irrespective of patient age. Clinical heterogeneity related to age was considered during the interpretation of the findings. Published systematic reviews and meta-analyses were used only to identify additional primary studies through citation screening and were not included in either the qualitative or quantitative synthesis, thereby avoiding duplication of evidence.

#### 2.4.2. Exclusion Criteria

Studies were excluded if they involved the topical, ophthalmic, or local use of an antimicrobial. Studies that were lacking clearly defined clinical endpoints, studies that evaluated prophylactic antimicrobial use rather than for therapeutic purposes, and studies that did not present quantitative data that could be extracted for a specific route of administration or specific outcome(s) were also not included in the quantitative meta-analysis.

### 2.5. Study Selection Process

**Figure 1:**
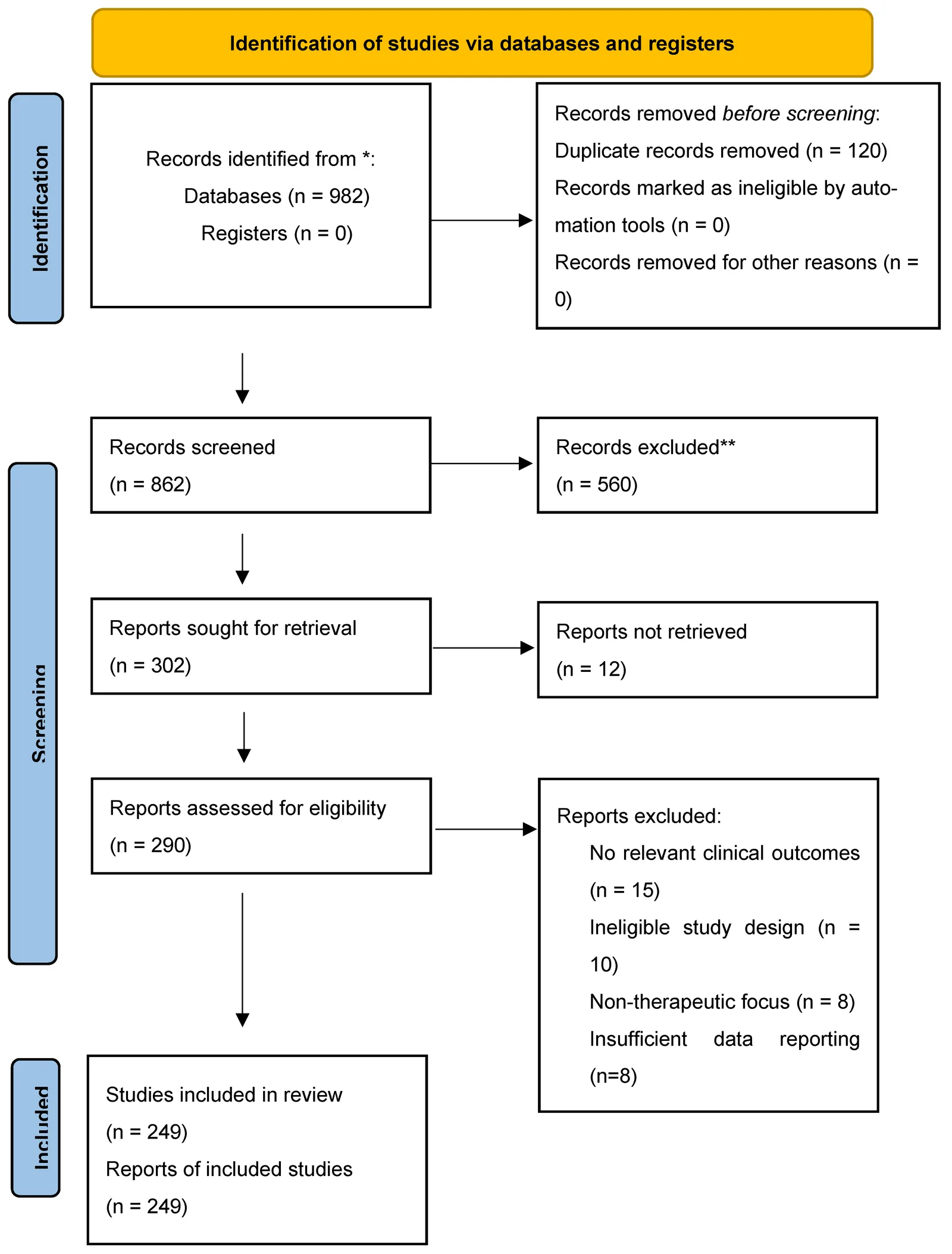
PRISMA flow diagram.

All the records of the database search were pooled and screened for eligibility. A total of 982 records were identified. The selection of the studies was conducted in a stepwise manner, starting with removing duplicate records and screening the title and abstract to identify potentially relevant studies. Then, the full-text articles were assessed against the eligibility criteria. After reading the full-text, 249 studies were found to be eligible and were included in the final analysis. Reasons for exclusion at the full-text stage were recorded. The process of study selection is outlined in the PRISMA flow diagram (Figure 1).

### 2.6. Data Extraction

A standardized and pre-defined data extraction form that was created in Microsoft Excel was used for data extraction. Extracted data included study characteristics like author, year of publication, study design, and setting, and population characteristics like sample size and mean age. Clinical variables were also recorded, such as the site of infection and follow-up period.

Antimicrobial intervention details (type of treatment and route of administration) were gathered with reported outcomes (clinical success, mortality, microbiological outcomes, and pharmacokinetic/pharmacodynamic parameters). Additional information, like statistical outcomes and variables that were adjusted in the analysis, was also gathered. The process of data extraction was conducted systematically to minimize inconsistencies and incomplete data in the extracted data from all the included studies. For studies that were part of the quantitative synthesis, other data extracted included treatment and control event counts, total sample sizes, outcome definitions, route of administration, and direction of effect for pooled meta-analysis calculations.

### 2.7. Quality Assessment Methods

The methodological quality and risk of bias of studies included were evaluated using the National Heart, Lung, and Blood Institute (NHLBI) Study Quality Assessment Tool.

This evaluation assessed various domains, such as adequacy of randomization, allocation concealment, based on these criteria, baseline comparability of study groups, loss of follow-up, adherence to intervention procedures, validity and reliability of outcome measures, adequacy of sample size, and use of intention-to-treat analysis. According to these criteria, all studies were classified as good, fair, or poor quality.

### 2.8. Data Synthesis

The studies were highly varied in their study designs, patient populations, administered antimicrobial therapies, and outcomes reported, and therefore, the synthesis was mostly qualitative (narrative). Only a subset of these studies was suitable for quantitative meta-analysis of the clinical and microbiological endpoints.

Selected studies were quantitatively synthesized based on the available numerical treatment and control data and similar outcome definitions. The clinical outcomes were clinical cure or clinical success (at specific evaluation time points), and the microbiological outcomes were pathogen eradication or presumed eradication.

For studies that did not report quantitative data or heterogeneous methodologies, non-comparable outcome definitions, or limited quantitative data, narrative synthesis was the primary method of analysis.

### 2.9. Statistical Analysis

Studies with standardized and extractable outcome data were included in the quantitative meta-analysis. Pooled effect estimates were used to synthesize clinical and microbiological outcomes. Fixed-effect and random-effects models were used as appropriate. Cochrane’s Q statistic and H^2^ values were used to evaluate the heterogeneity of the studies. In order to visualize the pooled treatment effects and the associated 95% confidence intervals, forest plots were created.

The funnel plot analysis was used to assess publication bias, as was Egger’s regression test. Furthermore, a network meta-analysis was conducted to evaluate the relative treatment effects between antimicrobial treatments and to compare the antimicrobial treatment strategies across different studies. Heterogeneity in study design, patient population, outcome definitions, and antimicrobial regimens led to the interpretation of treatment rankings with caution.

Software and computational environment. Bayesian network meta-analysis was performed using Python version 3.11.1 (64-bit, Windows 10 environment). Bayesian modelling and Markov chain Monte Carlo sampling were conducted using PyMC version 5.28.5, with PyTensor version 2.38.3 as the computational backend. Posterior inference and convergence diagnostics were assessed using ArviZ version 0.19.0. Data processing and statistical computations were performed using NumPy version 2.0.2, Pandas version 2.2.3, SciPy version 1.15.1, and Statsmodels version 0.14.1. Data visualization was performed using Matplotlib version 3.10.0 and Seaborn version 0.13.2. Excel file handling was performed using OpenPyXL version 3.1.2.

### 2.10. Ethical Considerations

Ethical approval was not sought for this research as it relied solely on data from previously published research, and no human or animal subjects were included.

## 3. Results

### 3.1. Study Selection

Database searching identified a total of 982 records. After screening the titles and the abstracts, potentially relevant studies were identified to be screened in the full text. A total of 249 studies were included in the qualitative synthesis. Of these, 34 studies fulfilled the predefined eligibility criteria for quantitative synthesis by reporting comparable outcomes and sufficient numerical data for meta-analysis. A sub-group of studies with quantitative eligibility criteria was selected and included in the clinical and microbiological outcomes meta-analysis. The study selection process and reasons for exclusion are presented in the PRISMA flow diagram (Figure 1).

### 3.2. Study Characteristics

The 249 included studies represented a heterogeneous body of evidence in terms of design, population, and clinical context. Most of them were randomized controlled trials (RCTs) (*n* = 112), followed by observational studies (*n* = 57) and other study designs (*n* = 80), such as pharmacokinetic/pharmacodynamic (PK/PD) studies, non-randomized studies, and other primary clinical investigations.

The sample sizes of the studies were also highly variable, with 3 to 12,706 patients included, as some of them were exploratory studies, and some were large cohorts [9]. This variation contributed to differences in statistical power and generalizability between studies to a large degree.

The clinical focus was mainly on respiratory infections, especially in populations that had cystic fibrosis and bronchiectasis. Pneumonia, such as community-acquired, hospital-acquired, and ventilator-associated pneumonia, was commonly researched. The additional infection sites were bloodstream infections, intra-abdominal infections, skin and soft tissue infections, urinary tract infections, and sepsis-related infections. Less frequently reported were otolaryngological, ocular, bone, central nervous system, and device-associated infections. In general, the prevalence of types of infections underscores the substantial concentration of data in pulmonary disease, with relatively less information in non-respiratory infections [10].

### 3.3. Antimicrobial Treatment Strategies

The variety of antimicrobial agents was examined in a large number of studies, and the use of anti-pseudomonal agents was a general trend. Most commonly reported antibiotics were tobramycin, ciprofloxacin, colistin (colistimethate sodium), piperacillin/tazobactam, ceftazidime, meropenem, and aztreonam, with extra use of carbapenems like imipenem and cefepime (Supplementary Table S1).

The treatment options in the studies were quite different and included both monotherapy and combination therapy. Inhaled antibiotics like tobramycin played a prominent role in respiratory infections, especially cystic fibrosis, in chronic or suppressive therapy [11,12,13]. Conversely, systemic treatments like beta-lactams and polymyxins were more commonly used in severe infections like bacteremia and those occurring in ICUs [14,15].

In general, there was evidence that the choice of treatment was dependent on the site of infection, severity, and antimicrobial resistance patterns, with an increasing trend toward the use of combination regimens (Supplementary Table S1) in the studies on multidrug-resistant *Pseudomonas aeruginosa*, even though clinical and microbiological outcomes were not consistent across studies [16,17].

### 3.4. Reported Outcomes

The findings and outcome definitions varied across studies and were extremely heterogeneous. A summary of the outcome definitions, measurement methods, and principal findings across the included studies is presented in Table 2. These were categorized into clinical, microbiological, functional, pharmacological, and safety in general (Supplementary Table S1 and S2).

The most reported outcomes were clinical outcomes, which consisted of clinical cure or treatment success, mortality, outcome of symptom resolution, and healthcare resource utilization (length of hospital or ICU stay). The success rates of the various studies were highly variable, with most of them being moderate to high, depending on the intensity of infection and treatment [18,19]. Mortality rates were more common among the critically ill, or more specifically, in the case of bloodstream or ICU-related infections [16,20].

Microbiological outcomes were bacterial eradication, culture negativity, and changes in bacterial load. Eradication showed inconsistent eradication rates between studies, which were high in the short-term eradication in some respiratory studies [21,22], but not in the long-term eradication. It also had some research that reported a change in minimum inhibitory concentration (MIC) that had fluctuating resistance patterns [23].

The functional outcomes of the respiratory system were the parameters of lung function that involved forced expiratory volume in one second (FEV1) and forced vital capacity (FVC). It was observed that the antimicrobial therapy, and especially inhaled therapy, was associated with positive effects [24,25], and the magnitude and durability of the changes were different among the studies.

Pharmacological outcomes (also reported) were PK/PD-related and were in AUC/MIC ratios, time above MIC (T>MIC), and target attainment. It has been mentioned in the articles that the populations of patients were very different in their exposure to drugs and target attainment, which means that there could be implications for treatment effectiveness and variability in clinical outcomes [26,27].

The adverse events of antimicrobial treatment were the safety outcomes. Most frequently, nephrotoxicity and ototoxicity were most frequently associated with the aminoglycosides and the polymyxins, including colistin [28,29].

Other endpoints included recurrence or relapses, time to exacerbation, inflammatory biomarkers (e.g., CRP, IL-6, TNF-alpha), quality of life scales, and biofilm responses. Overall, the outcome landscape was highly heterogeneous, and the most common endpoints were clinical response, microbiological eradication, lung function, mortality, and PK/PD target achievement.

### 3.5. Quality Assessment

The methodological quality in the included studies was highly heterogeneous. The NHLBI quality assessment tool indicated that 81 articles were of good quality, 75 of fair quality, and 83 of poor quality, which is a rather balanced distribution of study quality.

Reports of randomization, lack of blinding, and lack of consistency in the definition and measurement of outcomes were the common methodological weaknesses. Smaller studies, non-randomized design, were considered to be of lower quality and therefore were various observational and preliminary studies that were considered in this study [30,31]. This difference in quality of studies raised concerns about the overall quality of the evidence and lowered the comparability of studies.

### 3.6. Synthesis of Evidence

**Figure 2:**
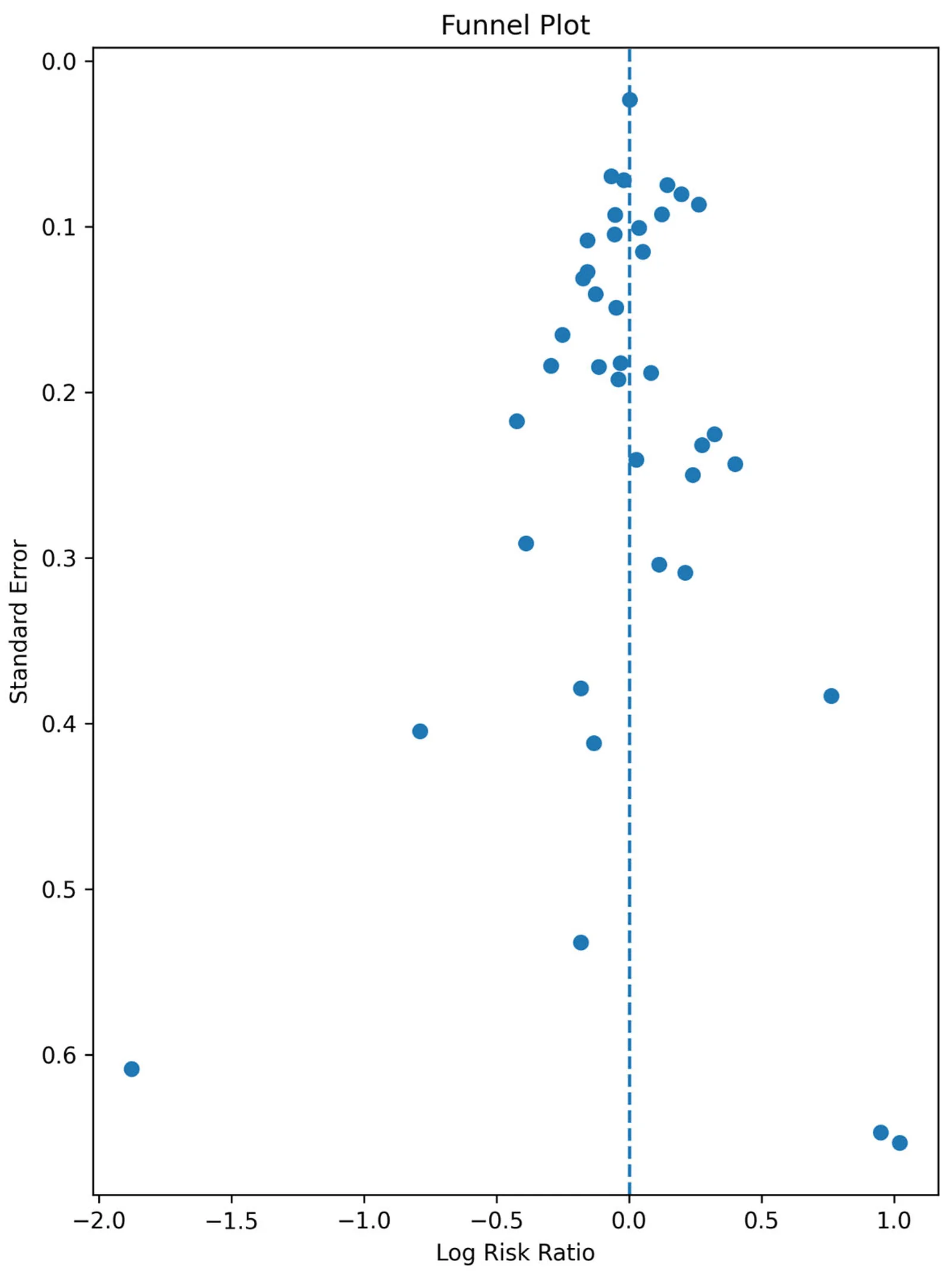
Funnel plot assessing publication bias among studies included in the quantitative meta-analysis.

The body of evidence lacked consistency regarding the study design, patient populations, antimicrobial interventions, and outcomes. Most of the literature was randomized controlled trials, but most of the literature was not only small but also specific to the subpopulation, i.e., cystic fibrosis patients, and therefore limited its application to a broader clinical population [32,33]. Publication bias among studies included in the quantitative synthesis was assessed using a funnel plot (Figure 2).

**Figure 3:**
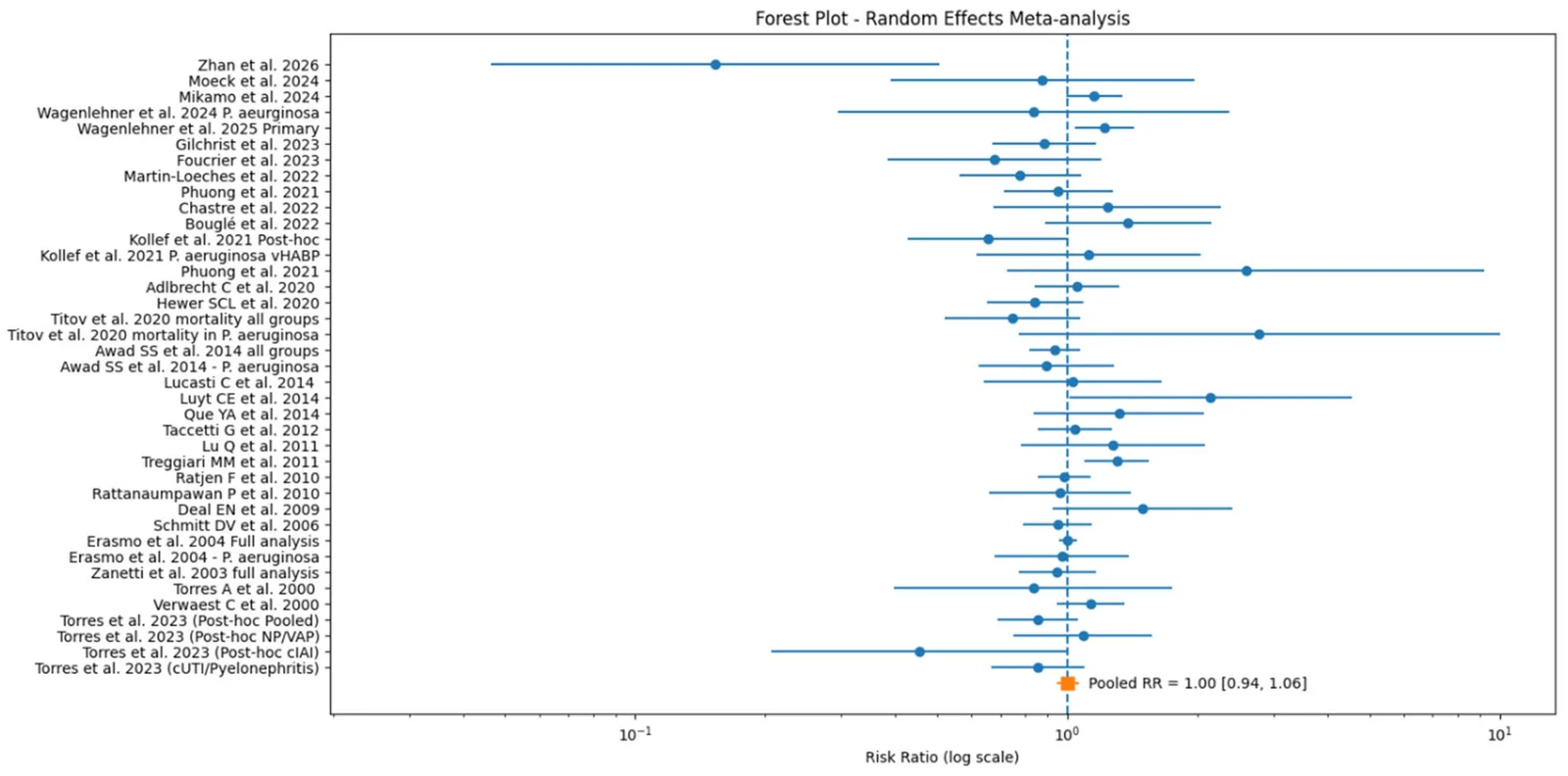
Forest plot. Cochran Q: 69.6154. Degrees of freedom: 38, H^2^: 1.8320. With fixed-effect model: Fixed RR: 1.0090; 95% CI: [0.9777, 1.0413]. With random effect model: Random RR: 1.0018; 95% CI: [0.9444, 1.0628]; EGGER TEST Intercept: −0.1197; *p*-value: 0.5838 [15,17,18,34,35,36,37,38,39,40,41,42,43,44,45,46,47,48,49,50,51,52,53,54,55,56,57,58,59].

Direct comparisons across the studies were also difficult because of the overall predominance of respiratory infections and the inconsistency in reporting the treatment regimens and outcomes. In addition, the inclusion of heterogeneous study designs, like non-randomized research and PK/PD research, also introduced methodological heterogeneity. There was significant heterogeneity in the results of all included studies (Table S2). The pooled treatment effects and heterogeneity statistics from the quantitative meta-analysis are presented in Figure 3.

**Figure 4:**
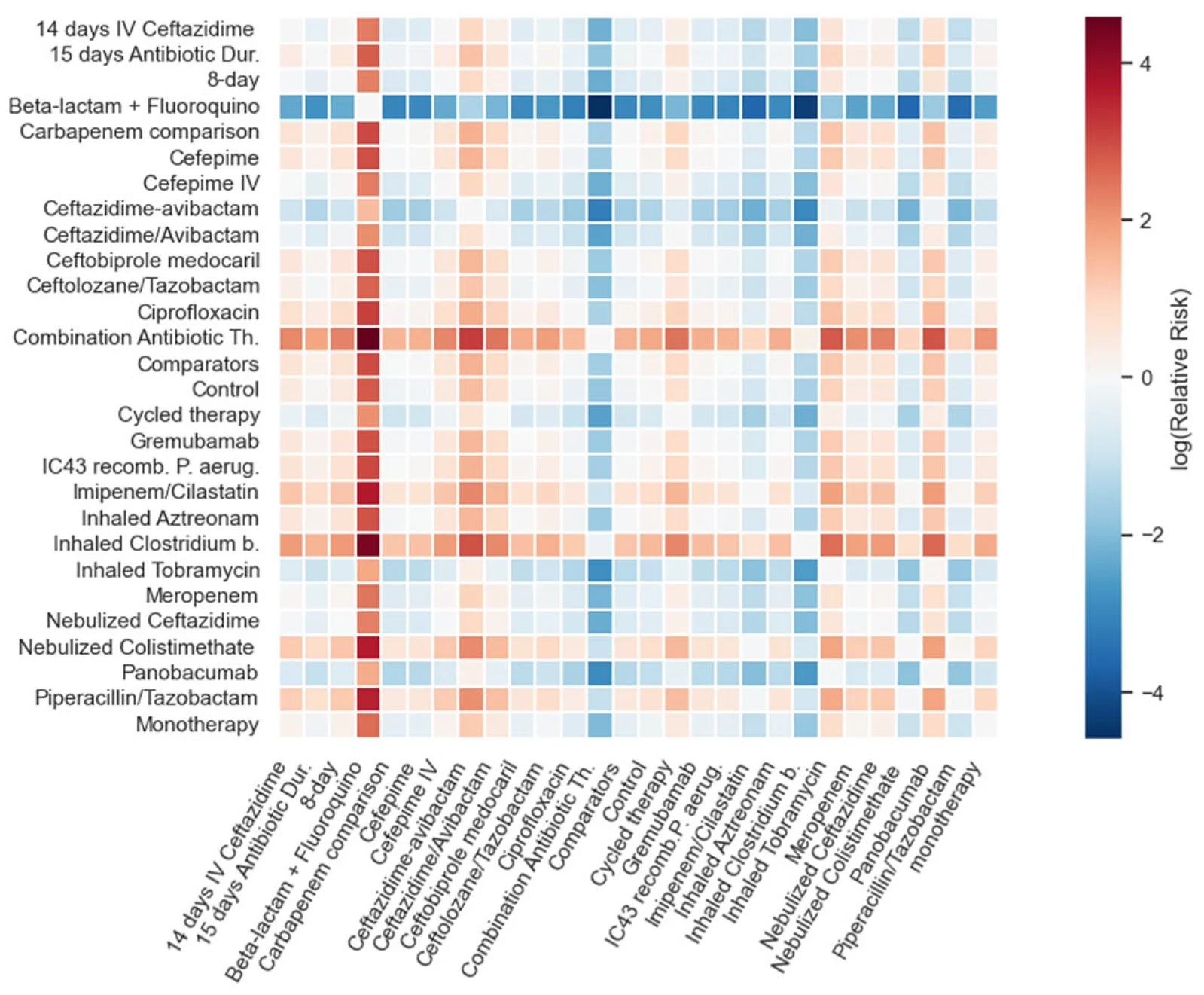
League table from the network meta-analysis showing relative treatment effect estimates across antimicrobial interventions.

To provide further evidence, we conducted a network meta-analysis. Based on the network meta-analysis, Combination Antibiotic therapy [17] study in COPD patients) showed the most favorable estimated treatment effect (d_mean = −1.89), followed by Inhaled Clostridium butyricum (d_mean = −1.63) and Imipenem/Cilastatin (d_mean = −0.96). Treatments such as Panobacumab (d_mean = 1.00) and Ceftazidime-avibactam versus comparator (d_mean = 1.27) demonstrated less favorable point estimates. However, interpretation should consider the associated uncertainty intervals, as treatment rankings based solely on point estimates may not reflect statistically significant differences. The league table generated from the network meta-analysis is presented in Figure 4. Estimated treatment effects relative to comparator treatments are summarized in Table 3.

**Figure 5:**
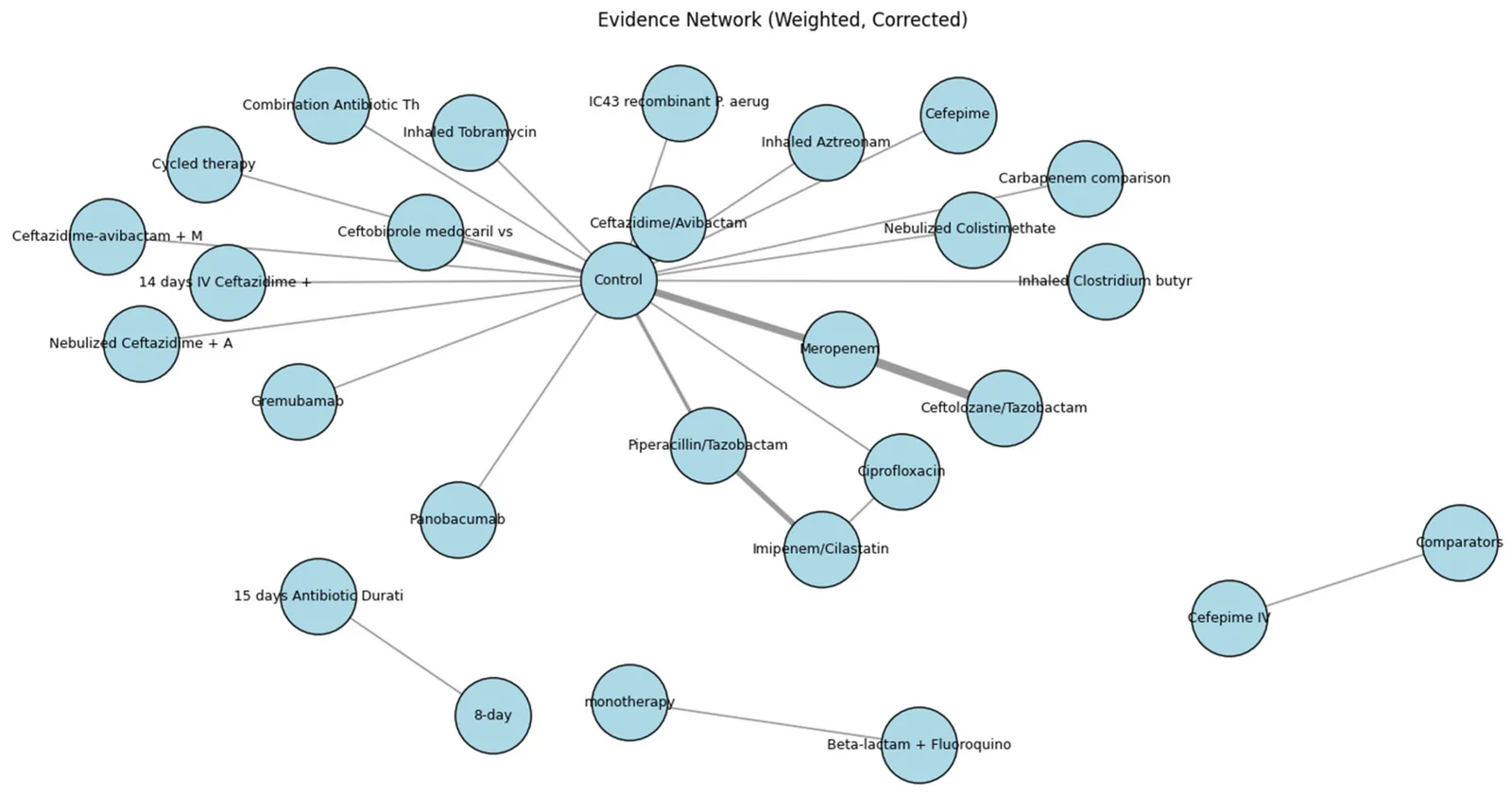
Treatment network illustrating direct and indirect comparisons among antimicrobial interventions included in the network meta-analysis. Nodes represent antimicrobial interventions, while connecting lines represent direct comparisons between treatments. Thicker lines indicate comparisons supported by a greater amount of evidence (weighted comparisons).

The network of treatment comparisons included in the network meta-analysis is illustrated in Figure 5.

However, a group of studies with standardized and extractable outcomes was chosen to be included in quantitative meta-analysis. Overall, the available evidence suggests that treatment effectiveness varies across clinical settings, and no antimicrobial regimen consistently demonstrated superiority across the diverse patient populations and infection types evaluated. However, this conclusion should be interpreted cautiously because of the substantial clinical and methodological heterogeneity and the variable quality of the included studies.

Since the primary analysis and synthesis of evidence showed a high heterogeneity between the studies, we decided to conduct two separate sub-analyses.

**Figure 6:**
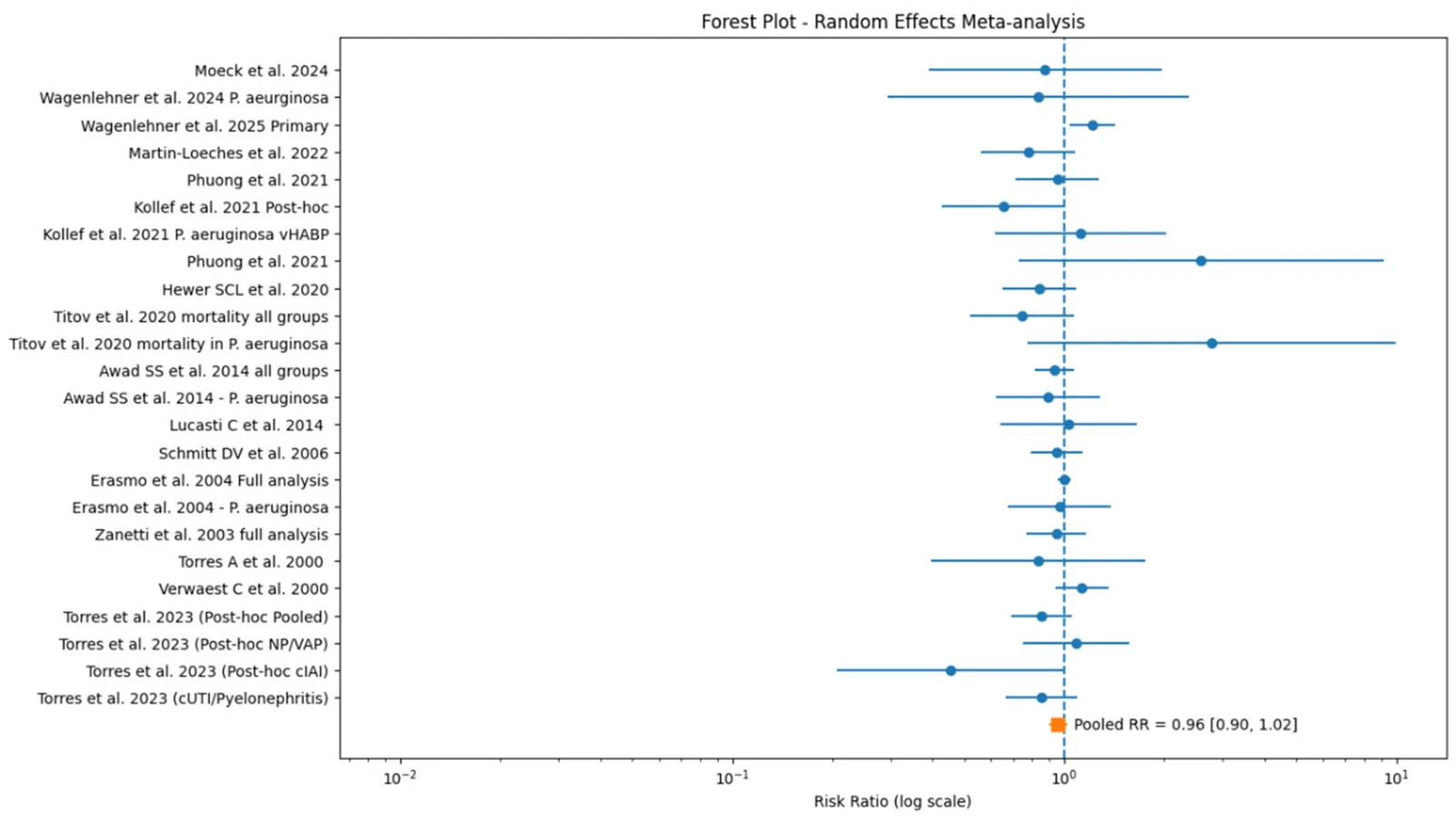
Forrest plot meta-analysis of IV therapies [15,17,18,35,37,40,43,45,46,54,55,56,57,58].

**Figure 7:**
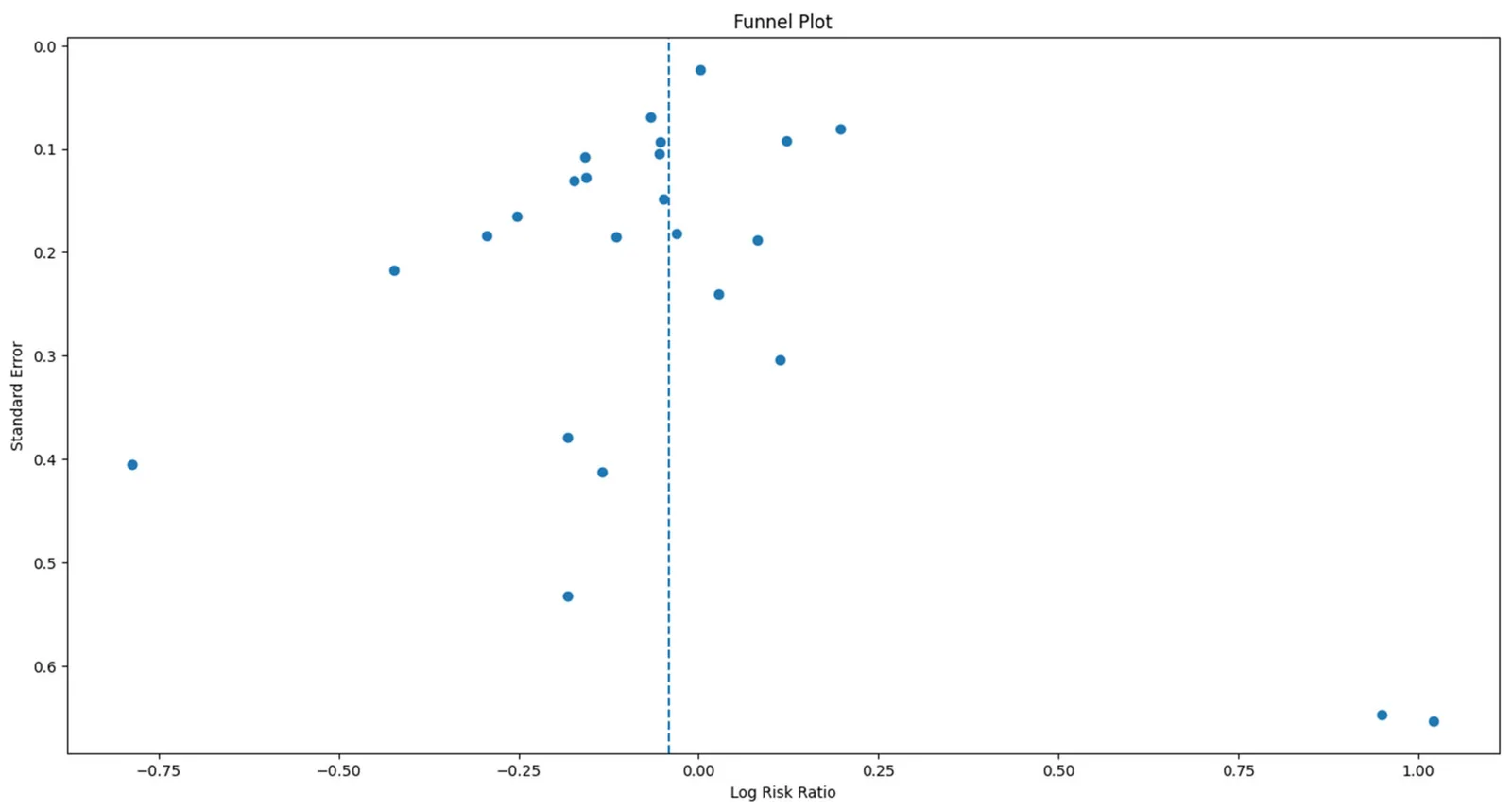
Funnel plot of IV therapies. Each point represents an individual study. The blue dashed vertical line indicates the pooled effect estimate (log risk ratio), which serves as the reference for assessing the symmetry of the funnel plot and potential publication bias.

As a first sub-analysis, we conducted a meta-analysis and network meta-analysis within the subset that includes only studies that tested the IV antimicrobial therapies, had a control group, and compared a clinically relevant endpoint with direct patient benefit. This meta-analysis, including 24 studies, was performed to evaluate the pooled relative risk (RR). Using a random-effects model, the overall pooled RR was 0.96 (95% CI: 0.90–1.02), indicating no statistically significant association between the exposure and outcome. The fixed-effect model produced a similar estimate (RR: 0.99, 95% CI: 0.95–1.02), supporting the robustness of the findings across modelling approaches. Between-study heterogeneity was low to moderate (τ^2^ = 0.0051; I^2^ = 29.13%), with Cochran’s Q test suggesting no significant heterogeneity (Q = 32.45, df = 23). These results indicate acceptable consistency across included studies. Assessment of publication bias using Egger’s regression test did not indicate significant asymmetry (intercept = −0.39, *p* = 0.73), suggesting no strong evidence of small-study effects. Overall, the findings indicate a neutral association with limited heterogeneity and no detectable publication bias. The corresponding forest plot for the meta-analysis of IV therapies is presented in Figure 6 with Funnel plot (Figure 7). 

**Figure 8:**
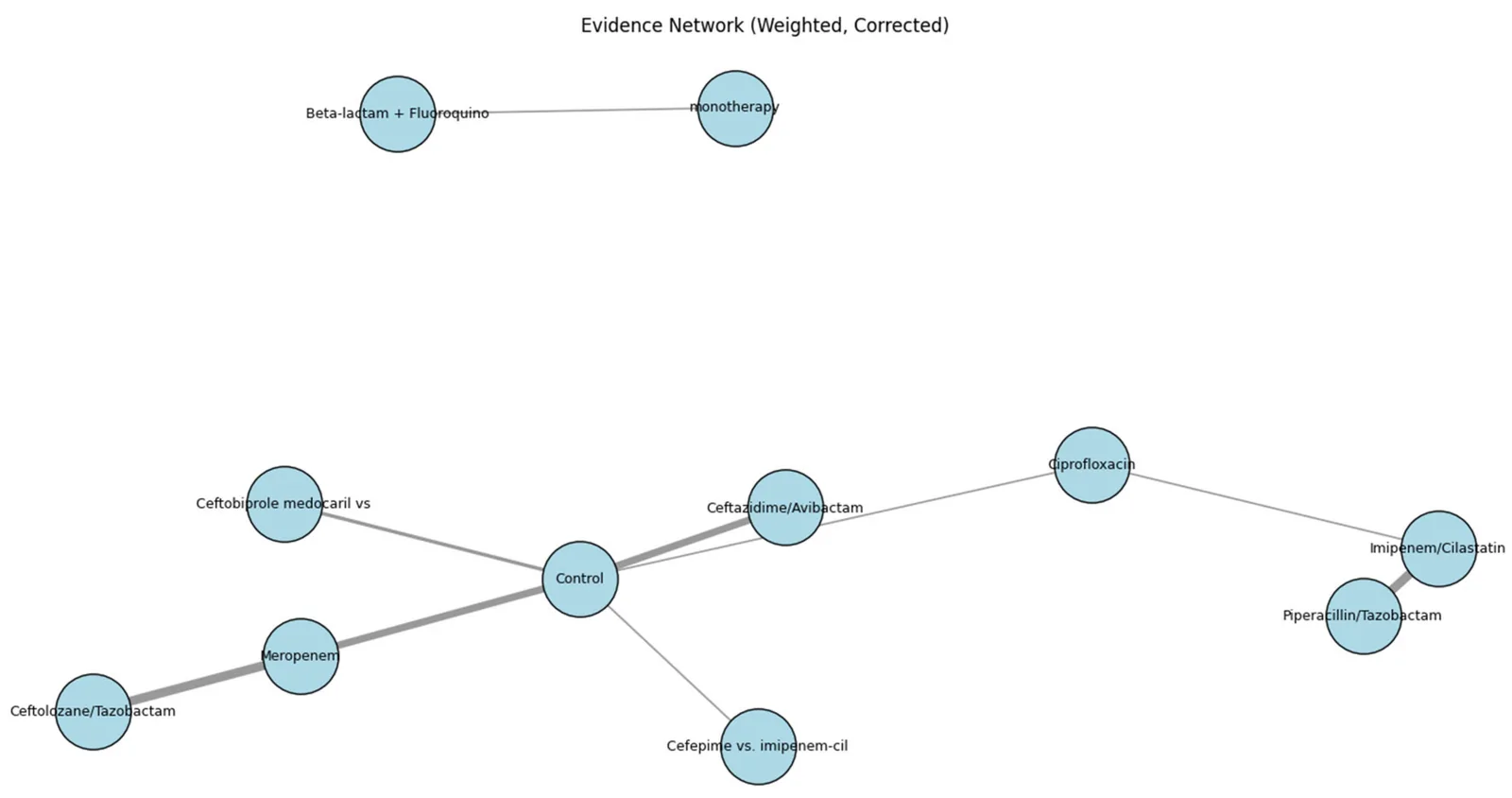
Network meta-analysis of IV therapies. Nodes represent individual antimicrobial interventions, while connecting lines represent direct comparisons between treatments included in the network meta-analysis. Thicker lines indicate comparisons supported by a greater amount of evidence (weighted comparisons).

The Bayesian random-effects network meta-analysis incorporating 22 studies and 15 antimicrobial regimens demonstrated broadly comparable efficacy across treatments for *Pseudomonas aeruginosa* infections. The estimated relative treatment effects showed considerable overlap, with all 95% highest density intervals crossing the null value, indicating no statistically credible evidence that any regimen was superior to the reference treatment. Between-study heterogeneity was low to moderate (τ = 0.279; 95% HDI 0.034–0.625), suggesting that treatment effects were generally consistent across the included studies despite differences in patient populations and clinical settings. Ranking analysis identified cefepime/taniborbactam as having the highest probability of being the most effective treatment (SUCRA = 0.810), followed by meropenem (SUCRA = 0.683) and carbapenem comparator regimens (SUCRA = 0.662), whereas ceftazidime/tobramycin (SUCRA = 0.304) and ceftazidime/avibactam (SUCRA = 0.335) ranked lowest. However, because the posterior credible intervals substantially overlapped and no statistically significant differences were observed between treatments, these rankings should be interpreted as probabilistic rather than definitive measures of comparative efficacy. Furthermore, the Bayesian model exhibited convergence issues, including 217 divergent transitions, an elevated R-hat for the heterogeneity parameter, and a low effective sample size for τ, indicating uncertainty in the posterior estimates and suggesting that the treatment rankings should be interpreted with caution. Overall, the findings indicate that currently available anti-pseudomonal regimens provide broadly similar clinical outcomes, with no convincing evidence supporting the superiority of any single antimicrobial therapy. The corresponding network meta-analysis of IV therapies is presented in Figure 8.

**Figure 9:**
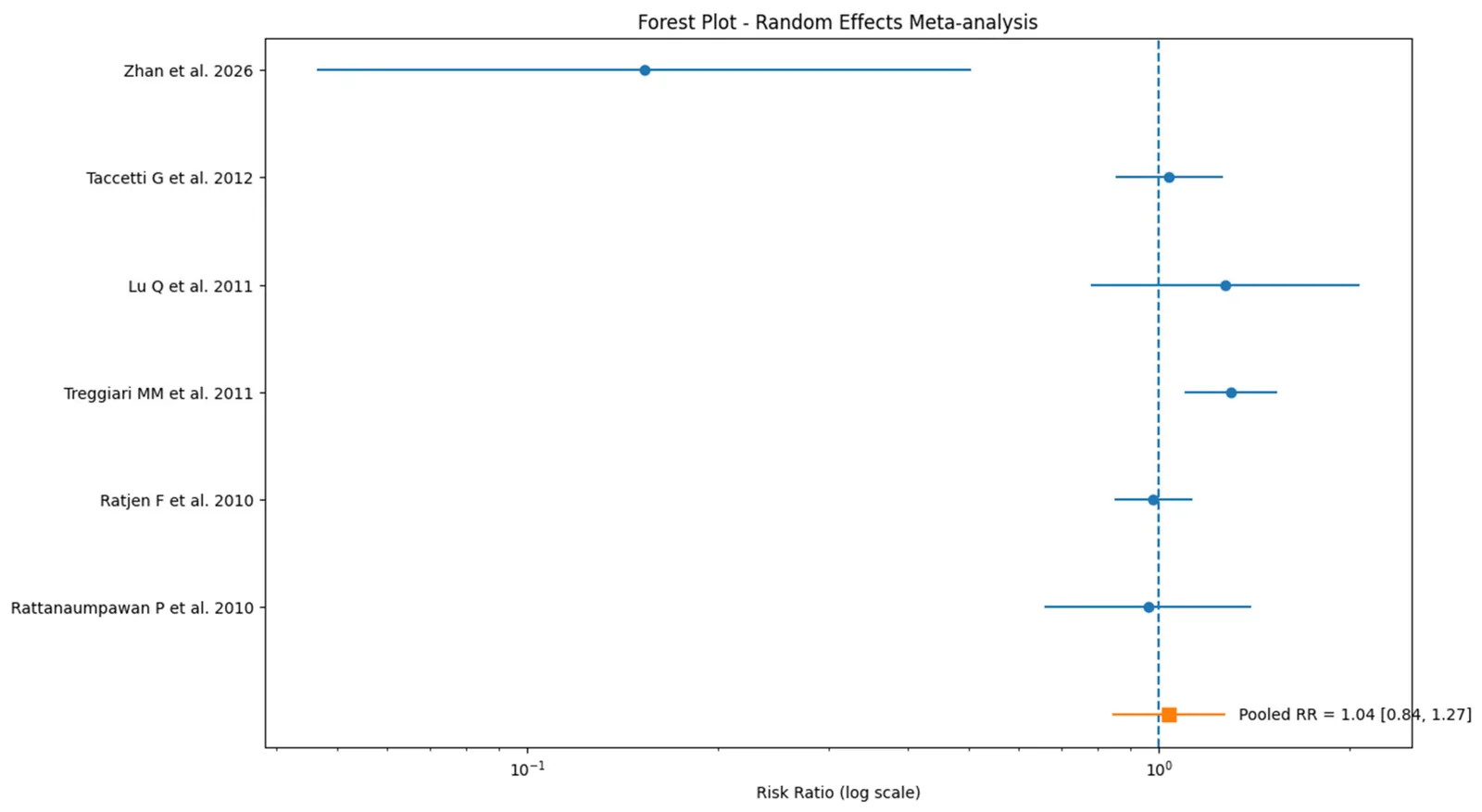
Forrest plot meta-analysis of inhaled therapies [34,48,49,50,51,52].

**Figure 10:**
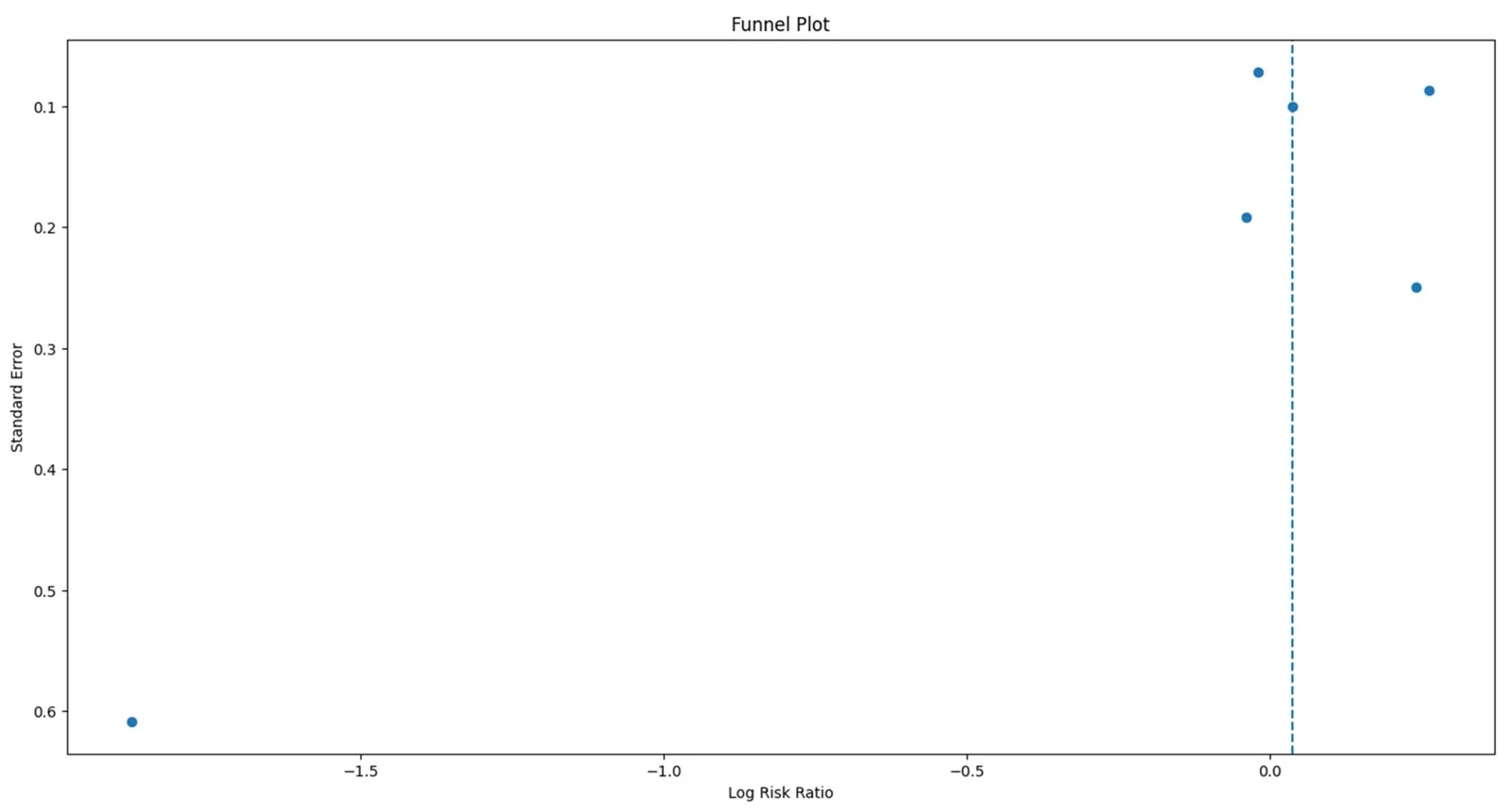
Funnel plot meta-analysis of inhaled therapies. Each point represents an individual study. The blue dashed vertical line indicates the pooled effect estimate (log risk ratio), which serves as the reference for assessing the symmetry of the funnel plot and potential publication bias.

As the second sub-analysis, we also separated studies with inhaled antimicrobials. A total of 6 studies were included in the meta-analysis. The pooled random-effects risk ratio (RR) was 1.037 (95% CI: 0.845–1.273), indicating no statistically significant association between exposure and outcome. Substantial heterogeneity was observed (I^2^ = 71.64%, τ^2^ = 0.0381; Cochran’s Q = 17.63, df = 5), suggesting notable between-study variability. The fixed-effect model produced a similar estimate (RR = 1.071, 95% CI: 0.979–1.172). Sensitivity analysis using leave-one-out procedures showed stable results, with pooled estimates ranging from 0.969 to 1.090, indicating no single study disproportionately influenced the overall effect. Egger’s test did not indicate significant publication bias (intercept = −1.634, *p* = 0.256). The 95% prediction interval (0.672–1.601) further reflected uncertainty and heterogeneity across potential future studies. Overall, the findings do not support a statistically significant association, though heterogeneity suggests underlying differences across studies. The corresponding forest plot for the meta-analysis of inhaled therapies is presented in Figure 9 and funnel plot for the meta-analysis of inhaled therapies is presented in Figure 10.

The strongest effect was observed in a [34] study with Oxygen-driven nebulization of *Clostridium butyricum* for *P. aeruginosa* pneumonia prevention. However, this effect was only observed in a single multicenter, randomized, single-blind, parallel-group, controlled study design, and, to the best of our knowledge, has not yet been replicated.

## 4. Discussion

The systematic review provides a synthesis of the evidence in a large and methodologically heterogeneous literature base and shows that the management of *Pseudomonas aeruginosa* infections is a complex problem. Despite the presence of antimicrobial classes and several randomized controlled trials, there is evidence that suggests similar efficacy of each of the treatment approaches. Instead, the impact of treatment appears to be quite circumstantial and dependent on the site of the infection, host factors, antimicrobial exposure, and adaptive capacity of the pathogen.

The disproportionate focus on respiratory infections, in particular, in the cohort of cystic fibrosis patients is one of the most notable features of the existing evidence base that facilitates interpretation but limits generalizability. Small alterations in the functioning of the lungs have been demonstrated to be associated with reductions in bacterial density and inhaled antibiotic treatment (mainly tobramycin and aztreonam) in numerous randomized trials [13,25,60,61]. They are not permanent, though, and do not generally result in lasting disease cure, as evidenced by recurrence rates and long-term follow-up [62,63]. This kind of separation is a serious problem in the treatment of *P. aeruginosa*: the ability to achieve temporary microbiological suppression, and not complete eradication.

Across comparative trials of treatment modalities, superiority hypotheses were generally not demonstrated. Therefore, in clinical trials involving intra-abdominal infections and ventilator-associated bloodstream infections, treatment outcomes can be expected to be broadly similar across treatment arms, partly because antimicrobial therapy alone does not always achieve cure as a stand-alone intervention.

Clinical cure rates of beta-lactam-based, carbapenem-based treatment, and even more recent drugs such as ceftolozane-tazobactam have similar clinical curative rates to randomized studies, yet demonstrate that the therapeutic limitations of *P. aeruginosa* infections are constrained by the drug of choice compared to biological and pharmacological factors, which limit effective bacterial eradication.

Combination regimens have been proposed to exhibit synergistic activity and suppress the emergence of resistance. After a careful review of the available evidence, we identified a study conducted by Arnaud Foucrier et al. (2023) [39]. In this study, the authors effectively addressed one of the key questions by demonstrating that monotherapy and combination antibiotic therapy were similar in terms of the recurrence rate of VAP, the incidence of extrapulmonary infections, and the acquisition of multidrug-resistant (MDR) bacteria during ICU stay. In this study, combination therapy consisted of a β-lactam plus an aminoglycoside or a fluoroquinolone, or a combination of a fluoroquinolone and an aminoglycoside. In contrast, definitive monotherapy was the use of a single active antimicrobial agent (a β-lactam, fluoroquinolone, or colistin), administered as directed antibiotic therapy throughout the entire treatment duration.

The first mechanistic explanation that can be offered for these observations of the limited efficacy of combination therapy is the heterogeneity of pharmacodynamics and pharmacokinetics of chronically infected and critically ill groups. Other authors observe that the interpatient variability of the beta-lactam exposure is highly variable, and even in the instance of a dose that is typically used, subtherapeutic concentrations are common [26,27]. Also, drug diffusion and biofilm-secreting respiratory secretions of the infected compartments are also postulated on impaired drug penetration despite the seemingly adequate systemic concentrations [64]. The reason behind the reduced drug activity can also be attributed to the fact that *P. aeruginosa* can form biofilm and adaptive resistance that results in phenotypic tolerance that cannot be detected by conventional susceptibility tests.

Another rather illustrative study that was excluded from sub-analyses due to being a prospective observational study was conducted [65]. In the study, it was concluded that imipenem-, meropenem-, and doripenem-treated patients had similar VAP recurrence rates (41%, 25%, and 22%, respectively; *p* = 0.15) and mortality rates (47%, 25%, and 22%, respectively; *p* = 0.07). Carbapenem resistance emerged similarly among patients treated with any carbapenem. No carbapenem was superior to another for preventing the emergence of carbapenem resistance. Our systematic review helps to draw very similar conclusions. That is, in the majority of cases, if *Pseudomonas aeruginosa* is susceptible to the prescribed antimicrobial, prescribing an additional antimicrobial for synergetic effects alone is unlikely to lead to significantly better outcomes. This conclusion also appears to extend to inhaled antibiotic strategies, although the evidence derives from a different clinical context and study design. For example, it was reported that no statistically significant differences between treatment strategies were observed and the odds ratios for *Pseudomonas aeruginosa*–positive cultures were 0.78 (95% CI, 0.49–1.23) for cycled versus culture-based therapy and 1.10 (95% CI, 0.71–1.71) for ciprofloxacin versus placebo, indicating no significant between-group differences [50].

It is also increasingly difficult to treat because of antimicrobial resistance. The most widespread treatments are not active, e.g., fluoroquinolones [23], which is why antimicrobial resistance becomes a major contributor to the problem of effective and timely treatment of the disease. Even in these cases, however, there will be no single solution by which the adaptive properties of the organism will be able to be fully overcome.

Interestingly, there are pathogen-directed novel interventions for the treatment of *Pseudomonas aeruginosa* infections. In the randomized study of 310 patients, inhaled Clostridium butyricum delivered via oxygen-driven nebulization significantly reduced the incidence of drug-resistant bacterial pneumonia compared with oral capsules (1.97% vs. 46.45%; RR 23.54, 95% CI 7.58–73.08). The inhaled formulation also led to improved clinical parameters and reduced respiratory bacterial colonization [34]. Another promising intervention is adjunctive immunotherapy with the anti-*Pseudomonas aeruginosa* LPS monoclonal antibody panobacumab. In a post hoc analysis of a phase IIa trial, patients receiving the full three-dose regimen demonstrated higher clinical resolution rates and a shorter time to recovery compared with those not treated with the antibody (85% vs. 64%; *p* = 0.048), despite having greater baseline disease severity [47].

However, in the EVADE phase 2 randomized controlled trial (*n* = 188), the bispecific monoclonal antibody MEDI3902 (gremubamab) did not reduce the incidence of nosocomial *Pseudomonas aeruginosa* pneumonia compared with placebo in mechanically ventilated, colonised patients (22.4% vs. 18.1%; *p* = 0.49). The estimated effect showed no significant relative risk reduction, and safety outcomes were comparable between groups [41]. In another randomized, double-blind phase 2/3 trial (*n* = 800), the IC43 *Pseudomonas aeruginosa* vaccine did not reduce 28-day all-cause mortality compared with placebo (29.2% vs. 27.7%; *p* = 0.67), nor did it significantly reduce infection-related outcomes [44].

There is substantial variability between studies that goes beyond study design: it is biological and clinical. They are all subject to variation, depending on host immunity, immune status, and differences in the location of the infection, and the length of the disease. This is also substantiated by the fact that most of the trials are cystic fibrosis-based and, therefore, could not have been generalized to an acute care setting, where the dynamics of the disease and the management priorities are extremely different. It is among the reasons why such a fragmented evidence base is bound to lead to the insufficiency of clear treatment recommendations.

The clinical environment outcomes allow for the refinement of conventional treatment algorithms into more personalized and precision-based approaches. It manifests itself and is reflected in the fact that the respiratory infection progresses more gradually, and the antibiotic therapy is initiated earlier [66], but it is also possible that it might be required to optimize the dosage, according to the principles of PK/PD. The influence of therapeutic drug monitoring on hard clinical results has been unclear despite the potential to increase target attainment [67], which explains why the necessity to formulate additional studies arises.

The review has strengths such as the breadth of included studies in the review and the synthesis of clinical, microbiological, and pharmacological evidence. The limitations of this review are, however, the substantial heterogeneity, variability in study quality, and inconsistency in outcome definitions. Due to heterogeneity, pooled analysis was not performed on all included studies, and selected studies with standardized outcome reporting were quantitatively analyzed, allowing for a better comparative interpretation of the clinical and microbiological outcomes.

The findings of this review should be interpreted in the context of several important methodological limitations. Although quantitative synthesis was restricted to studies with standardized and extractable outcome data, considerable clinical and methodological heterogeneity remained across the included studies with respect to patient populations, infection sites, antimicrobial regimens, outcome definitions, and study designs. Furthermore, the variable methodological quality of the included evidence may have influenced the precision and reliability of the pooled estimates. Consequently, the absence of a consistently superior antimicrobial regimen should not be interpreted as evidence of equivalent efficacy across all therapeutic strategies, but rather as reflecting the diversity of clinical settings and the limitations of the available evidence. These findings support the need for standardized outcome reporting, adequately powered randomized controlled trials, and harmonized methodological approaches to strengthen the evidence base for the management of *Pseudomonas aeruginosa* infections.

Further studies are required where the focus is on large multicentre randomized trials that balance the outcomes and other patient groups other than cystic fibrosis. The rapid diagnostics can be applied in combination with rapid resistance profiling, where the majority of optimal individualized treatment strategies can be developed. It will also involve the need to develop new antimicrobial agents and other alternative treatments against it, so that they can be capable of addressing the issue of antimicrobial resistance that is currently arising.

## 5. Conclusions

In conclusion, this systematic review reveals the fact that the treatment of *Pseudomonas aeruginosa* infections is a complex and context-specific challenge. The range of antimicrobial agents is extensive, and it does not imply that a particular approach to treatment is universally applicable; it might be more effective in various clinical settings. These combinations of factors, such as site of infection, host factors, antimicrobial resistance patterns, and variability in drug exposure, determine the outcome of treatment. Still, these gaps in evidence base and standardization of the study design and outcome continue to hinder the development of definitive treatment guidelines. Future progress may be supported by more individualized treatment approaches that integrate clinical, microbiological, and pharmacological factors that entail clinical, microbiological, and pharmacological factors. Quantitative synthesis did not demonstrate consistent evidence supporting the superiority of any antimicrobial regimen across the evaluated clinical settings; however, this finding should be interpreted in light of the clinical and methodological heterogeneity of the available evidence. Future research should prioritize harmonized outcome measures, adequately powered multicentre randomized controlled trials, optimization of antimicrobial dosing strategies, and rigorous evaluation of emerging therapeutic approaches to strengthen the evidence base for the management of *Pseudomonas aeruginosa* infections.

## Figures and Tables

**Table 1:** PRISMA-S checklist.

Section/Topic	#	Checklist Item	Location(s) Reported
INFORMATION SOURCES AND METHODS			
Database name	1	Name each database searched, stating the platform for each.	Section 2.2 and Section 2.3: MEDLINE via PubMed, Cochrane Library, Embase, Web of Science, and Scopus.
Multi-database searching	2	If databases were searched simultaneously on a single platform, state the name of the platform, listing all of the databases searched.	Section 2.3: Databases were searched individually; no simultaneous multi-database platform search is reported.
Study registries	3	List any study registries searched.	We searched for studies reported in research databases. Identified literature reviews were also screened for relevant studies.
Online resources and browsing	4	Describe any online or print source purposefully searched or browsed (e.g., tables of contents, print conference proceedings, websites), and how this was done.	Section 2.3: reference lists of included studies and relevant reviews were manually screened; no other online or print browsing sources are reported.
Citation searching	5	Indicate whether cited references or citing references were examined, and describe any methods used for locating cited/citing references (e.g., browsing reference lists, using a citation index, setting up email alerts for references citing included studies).	Section 2.2 and Section 2.3: cited references/reference lists of included articles and relevant reviews were manually screened.
Contacts	6	Indicate whether additional studies or data were sought by contacting authors, experts, manufacturers, or others.	Authors, experts, and manufacturers were not contacted during this study.
Other methods	7	Describe any additional information sources or search methods used.	Section 2.2 and Section 2.3: manual reference-list screening was used as an additional method.
SEARCH STRATEGIES			
Full search strategies	8	Include the search strategies for each database and information source, copied and pasted exactly as run.	Section 2.3.1: PubMed search string is reported; equivalent syntax adaptations for Embase, Web of Science, Scopus, and Cochrane Library are described but not copied in full.
Limits and restrictions	9	Specify that no limits were used, or describe any limits or restrictions applied to a search (e.g., date or time period, language, study design) and justify their use.	Section 2.3.2 and Abstract: English, German, and French; human studies; clinical trials, RCTs, meta-analyses, and systematic reviews; 1 January 2000–31 March 2026.
Search filters	10	Indicate whether published search filters were used (as originally designed or modified), and if so, cite the filter(s) used.	Section 2.3.2: database filters are reported where available, including PubMed Clinical Trial, RCT, Systematic Review filters, and Cochrane review filters; no published named search filter is cited.
Prior work	11	Indicate when search strategies from other literature reviews were adapted or reused for a substantive part or all of the search, citing the previous review(s).	Section 2.3.
Updates	12	Report the methods used to update the search(es) (e.g., rerunning searches, email alerts).	We re-ran the search strategy before completing the research from reviewed studies from 2000 to 2020. A repeated search for studies was also done before performing the data synthesis.
Dates of searches	13	For each search strategy, provide the date when the last search occurred.	Section 2.3.2 and Abstract: searches covered publications from 1 January 2000 to 31 March 2026; the last search date appears to be 31 March 2026.
PEER REVIEW			
Peer review	14	Describe any search peer review process.	All authors reviewed the processes that are described.
MANAGING RECORDS			
Total Records	15	Document the total number of records identified from each database and other information sources.	Abstract, Section 2.5 and Section 3.1: 982 total records identified; database/source-level counts are not reported.
Deduplication	16	Describe the processes and any software used to deduplicate records from multiple database searches and other information sources.	Section 2.5: Duplicate records were removed during stepwise screening; specific software and a detailed deduplication process are not reported.

PRISMA-S: An Extension to the PRISMA Statement for Reporting Literature Searches in Systematic Reviews. Rethlefsen ML, Kirtley S, Waffenschmidt S, Ayala AP, Moher D, Page MJ, Koffel JB, PRISMA-S Group. Last updated 27 February 2020.

**Table 2:** Summary of outcome definitions, measurement methods, and principal findings across the included studies.

Outcome Category	Outcome Definition	Measurement Method	Overall Findings
Clinical outcomes	Clinical cure, clinical success, symptom resolution, mortality, length of hospital/ICU stay	Investigator assessment, predefined clinical endpoints, follow-up evaluation	Clinical outcomes varied considerably across studies, with no antimicrobial regimen consistently demonstrating superiority.
Microbiological outcomes	Bacterial eradication, presumed eradication, culture negativity, changes in bacterial load	Culture results, microbiological assessment, MIC determination	Eradication rates were variable and influenced by infection type, antimicrobial susceptibility, and follow-up duration.
Functional outcomes	Lung function (FEV_1_, FVC)	Spirometry	Inhaled antimicrobial therapies generally improved pulmonary function in respiratory infections, although the magnitude and durability of benefit differed across studies.
Pharmacokinetic/Pharmacodynamic outcomes	AUC/MIC, T>MIC, target attainment	PK/PD analyses and therapeutic drug monitoring	PK/PD target attainment varied substantially among patient populations and may have influenced treatment effectiveness.
Safety outcomes	Nephrotoxicity, ototoxicity, adverse events	Clinical monitoring and adverse event reporting	Aminoglycosides and polymyxins were most frequently associated with treatment-related toxicity.
Other outcomes	Recurrence, relapse, time to exacerbation, inflammatory biomarkers, quality of life	Study-specific clinical and laboratory assessments	Outcome definitions were heterogeneous, limiting direct comparison across studies.

**Table 3:** Estimated treatment effects relative to comparator treatments from the network meta-analysis.

Treatment	d_Mean
Combination Antibiotic	−1.89
Inhaled Clostridium butyricum	−1.63
Imipenem/Cilastatin	−0.96
Nebulized Colistimethate	−0.90
Piperacillin/Tazobactam	−0.82
Ciprofloxacin	−0.44
Carbapenem comparison	−0.34
IC43	−0.33
Comparators	−0.31
Cefepime	−0.28
Inhaled Aztreonam	−0.25
Ceftobiprole medocaril vs. comp.	−0.24
Gremubamab	−0.23
Control	−0.13
15 days Antibiotic Duration	−0.07
Ceftolozane/Tazobactam	0.01
Monotherapy	0.12
Meropenem	0.25
14 days IV Ceftazidime vs. comp.	0.30
Cefepime IV	0.33
8-day	0.37
Nebulized Ceftazidime vs. comp.	0.37
Ceftazidime/Avibactam	0.57
Cycled therapy	0.60
Inhaled Tobramycin	0.91
Panobacumab	1.00
Ceftazidime-avibactam vs. comp.	1.27

## Data Availability

The data obtained, Excel data extraction, synthesis, and quantitative analysis are supplied in the Supplementary Materials attached to this article.

## Supplementary Materials

The following supporting information can be downloaded at https://www.mdpi.com/article/10.3390/life16081267/s1. Supplementary Materials. Additional data, statistical analyses, and computational resources supporting the findings of this study are provided. Supplementary File S1 (Analysis_V2.py) contains the Python code used for data processing, statistical analyses, and generation of quantitative synthesis outputs. Supplementary File S2 (meta_analysis_results.xlsx) provides the results of the conventional pairwise meta-analysis, including pooled effect estimates, confidence intervals, and heterogeneity statistics. Supplementary File S3 (Network meta V2.py) contains the Python code used for Bayesian network meta-analysis, including model specification, Markov chain Monte Carlo sampling, posterior estimation, and treatment ranking procedures. Supplementary File S4 (NMA_report 4 chains.xlsx) presents the complete Bayesian network meta-analysis output generated using four independent Markov chain Monte Carlo chains, including posterior estimates, convergence diagnostics, and treatment comparison results. Supplementary File S5 (Quant_meta_V2.xlsx) provides the quantitative meta-analysis dataset used for statistical analyses, including extracted study-level characteristics, interventions, comparators, and outcome data. Supplementary File S6 (Table S1. Selected_studies_review.xlsx) summarizes the characteristics of studies included in the systematic review. Supplementary File S7 (Table S2.xlsx) contains additional extracted study-level variables and outcome data supporting the quantitative synthesis and network meta-analysis.

## Abbreviations

The following abbreviations are used in this manuscript:

AUC/MIC	Area Under the Curve to Minimum Inhibitory Concentration Ratio
CF	Cystic Fibrosis
CI	Confidence Interval
CRP	C-Reactive Protein
FEV1	Forced Expiratory Volume in One Second
FVC	Forced Vital Capacity
ICU	Intensive Care Unit
IL-6	Interleukin-6
INH	Inhalation
IV	Intravenous
MDR	Multidrug-Resistant
MIC	Minimum Inhibitory Concentration
NHLBI	National Heart, Lung, and Blood Institute
PK/PD	Pharmacokinetics/Pharmacodynamics
PO	Oral (Per Os)
PRISMA	Preferred Reporting Items for Systematic Reviews and Meta-Analyses
RCT	Randomized Controlled Trial
RR	Risk Ratio
T>MIC	Time Above Minimum Inhibitory Concentration
TNF-alpha	Tumor Necrosis Factor Alpha
XDR	Extensively Drug-Resistant
